# Risk map for wolf threats to livestock still predictive 5 years after construction

**DOI:** 10.1371/journal.pone.0180043

**Published:** 2017-06-30

**Authors:** Adrian Treves, Mark F. Rabenhorst

**Affiliations:** 1 Nelson Institute for Environmental Studies, University of Wisconsin–Madison, Madison, Wisconsin, United States of America; 2 Carnivore Coexistence Lab, Madison, Wisconsin, United States of America; Michigan Technological University, UNITED STATES

## Abstract

Risk maps are spatial models of environmental hazards such as predation on livestock. We tested the long-term validity of a published risk map built from locations where Wisconsin wolves attacked livestock from 1999–2006. Using data collected after model construction, we verified the predictive accuracy of the risk map exceeded 91% for the period 2007–2011. Predictive power lasting 5 years or more substantiates the claim that risk maps are both valid and verified tools for anticipating spatial hazards. Classification errors coincided with verifier uncertainty about which wolves might be responsible. Perceived threats by wolves to domestic animals were not as well predicted (82%) as verified attacks had been and errors in classification coincided with incidents involved domestic animals other than bovids and verifier uncertainty about which wolves were involved. We recommend risk maps be used to target interventions selectively at high-risk sites.

## Introduction

Risk maps are predictive models with spatial components that distinguish locations by the probabilities that an environmental hazard occurs there. Risk maps have attracted growing interest in various environmental and crime prevention fields [1–4], because the algorithms and spatial data needed to build and validate them have become more available and sophisticated. The sophistication has permitted investigators to verify that risk probabilities are valid and truly predictive. True verification using data that were not included in model construction remains a rare but valuable step for the external validation of risk maps.

**Fig 1:**
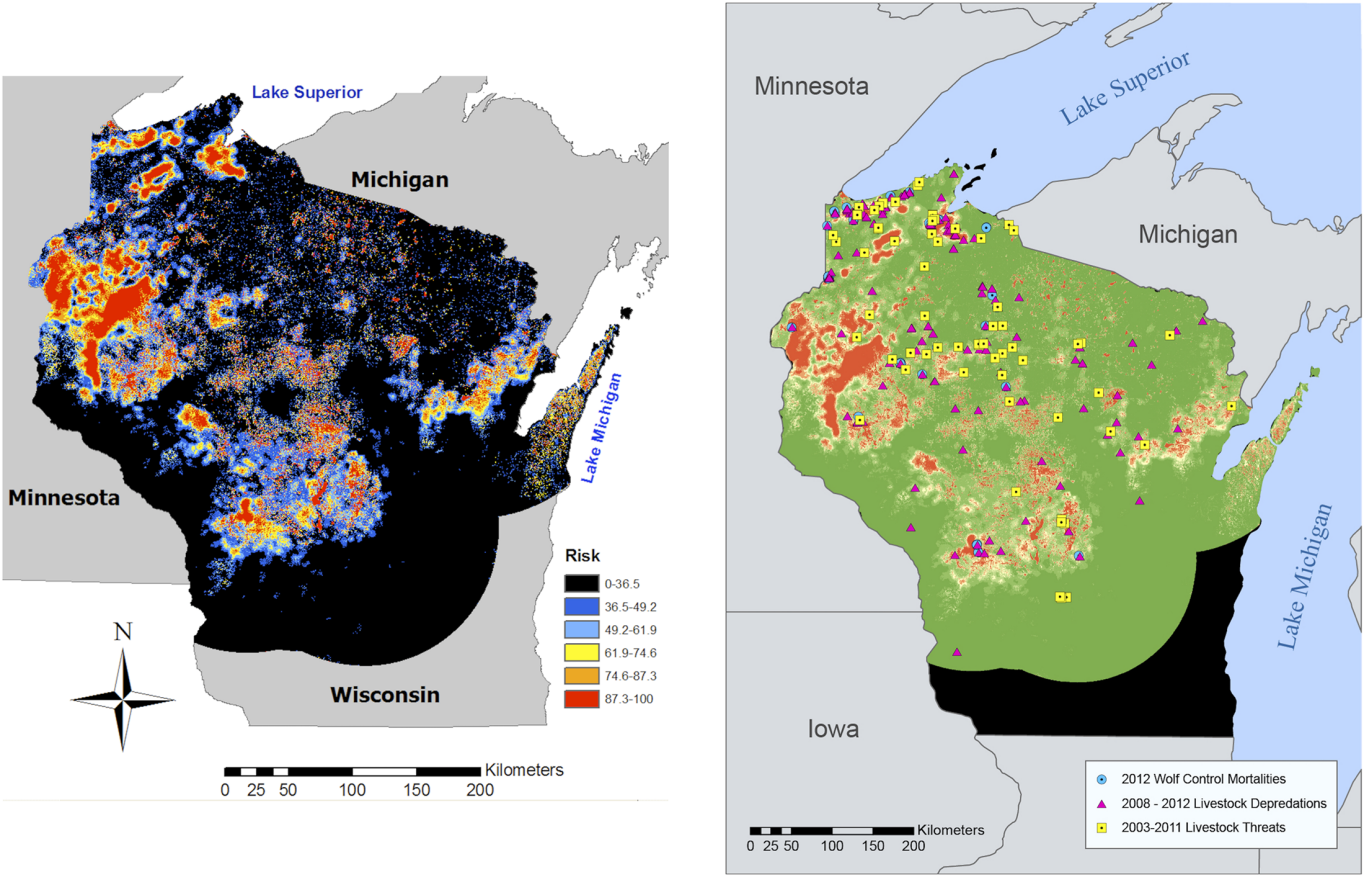
Risk map for wolf predation on livestock in Wisconsin. **A**: the analysis area (black) showing published risk probabilities in six categories [3]. **B**: Wisconsin and the analysis area (green) with risk colors dichotomized into high-risk (red), lower-risk (orange), and very low risk (green). Symbols depict locations of verified incidents of wolf attack on livestock (magenta triangles), perceived threats to livestock (yellow squares), and sites where the WDNR killed one or more wolves to prevent livestock loss 2012 (blue circles). Note that symbols are larger than the pixels colored by risk category, so they may obscure underlying risk levels.

Here we test the hypothesis that a risk map has predictive power up to 5 years beyond its construction date. We built a risk map of gray wolf (*Canis lupus*) predation on cattle using 133 verified attacks in Wisconsin, USA, from 1999–2006 [3]. In that publication, we verified that risk map with 60 additional incidents from 2007–2009, which had not been used in model construction (Fig 1A). The risk map correctly predicted 88% of those new sites were high-risk [3]. Here we take one step further to evaluate quantitatively if the risk map had predictive power through 2011, using 122 additional locations statewide, which accumulated after publication of [3]. We also examine patterns of errors.

## Methods

The methods for constructing the risk map were detailed in [3]. Here we mapped 122 new incidents into the existing risk map using ArcGIS 10. We then compared modeled risk value each new incident (0 for the lowest risk category and 1 for any other) and tested the proportion that were correct or erroneous classifications against chance probabilities based on expected proportions from the map as a whole, using Fisher’s exact test. We then calculated the relative frequencies of the six risk categories (colors in Fig 1A) assigned to each new incident and compared the observed frequencies to the expected frequencies of each risk category (color) across the whole map. We used that comparison qualitatively to assess if the risk map color scheme in Fig 1A was useful.

We then related prediction errors in the model (false negatives) to the type of livestock involved (bovid or other), size (l = larger than a typical calf or s = small) and to the certainty (? or no question mark in the verifier’s record) about which wolf pack was involved. We code certainty as “firm” when a field investigator implicated a specific wolf pack and as “unsure” when the investigator did not mention a pack or added a “?” in that field of the original Wisconsin Department of Natural Resources (WDNR) reports.

Finally we tested a different type of incident we refer to as perceived threats–defined as complaints arising from perceived wolf threats to livestock that did not result in injury or loss from 2003–2011 –using the same methods as above.

The source data are available at [5].

## Results and discussion

The published risk map correctly predicted that 92% of the 122 new sites from 2010–2011 would face some risk of an incident (Fisher’s exact P<0.001). Therefore, the underlying model retained strong predictive power for 13 years (1999–2011).

Regarding the validity of the categories of risk (the color scheme in Fig 1A that grouped risk probabilities into six categories, we found that 48% of all incidents fell into the highest risk pixels (red), which comprised 6.2% of the analysis area. The remaining 44% of correctly predicted sites fell more evenly into lower-risk pixels (orange–blue) that occupied 26.5% of the analysis area (Fig 1A). We concluded that three risk categories might be more valid than six categories. Accordingly we mapped the highest-risk category as red, all lower-risk categories as yellow, and the area predicted to be lowest risk as green in Fig 1B.

Considering all 182 incidents from 2007–2011 (after model construction [3]), 91% were correctly classified and errors in categorization of incidents coincided with uncertainty about which wolf pack was involved (n = 36 unsure, n = 146 firm: Fisher’s exact P = 0.007). Neither the size of the livestock (n = 34 l, n = 137 s, P = 0.53), nor the type of livestock (n = 163 bovid, n = 19 other, P = 1.0) predicted those errors, as previously thought [3].

We also tested the published model on 96 perceived threats to domestic animals on private properties (Fig 1B). The model predicted 82% of these locations correctly (P<0.01). Just over a quarter of threats fell in the highest-risk pixels (red), suggesting different phenomena might explain perceived threats than explained verified attacks. Errors were associated with perceived threats to non-cattle (n = 25 equids, ovids, canids, and people, n = 71 bovids, P<0.012). Among the cattle, errors in classification again coincided with uncertainty about which wolf pack was involved (n = 25 uncertain, n = 71 certain; P<0.012).

## Conclusions

A risk map built with data collected from 1999–2006 continued to accurately predict the locations that might risk wolf attacks on livestock, as late as 2011. Moreover the risk model situated almost half of all incidents in the highest-risk category of pixels, thereby usefully focusing attention on <7% of the statewide analysis area. All risky pixels summed to <33% of that same area. We infer that refocusing preventive action in that area comprising less than one-third of a state would improve management efficiency.

Complaints of perceived threat from wolves from 2003–2011 were 10% less predictable, possibly because these complaints involved a high proportion of non-cattle that are rarely attacked by wolves [6], or because perceived threats are an unreliable indicator of actual risk.

Despite the publication of three verified risk maps for Wisconsin, Minnesota, and Michigan demonstrating that wolves attacked livestock in highly predictable locations [3, 7, 8], state governments have not used these to protect the private interest in livestock and the public interest in wolves, to our knowledge. Instead, all three states articulated goals to reduce wolf populations and implemented reactive, lethal methods to achieve that goal to reduce livestock losses (Fig 1B). Given growing doubts about the effectiveness of killing carnivores to protect livestock [9, 10], we recommend investment in prevention at high-risk sites. Risk maps present a tool for such targeting.

## Supporting information

S1 Dataset (XLSX)
